# Effect of temporary housing on incidence of diabetes mellitus in survivors of a tsunami-stricken area in 2011 Japan disaster: a serial cross-sectional RIAS study

**DOI:** 10.1038/s41598-020-71759-4

**Published:** 2020-09-21

**Authors:** Shuko Takahashi, Kozo Tanno, Yuki Yonekura, Haruki Shimoda, Ryohei Sasaki, Kiyomi Sakata, Akira Ogawa, Seiichiro Kobayashi

**Affiliations:** 1 Division of Medical Education, Iwate Medical University, Idaidori 1-1-1, Yahaba-cho, Shiwa-gun, Iwate, Japan; 2 Takemi Program in International Health, Harvard T.H. Chan School of Public Health, Boston, MA USA; 3 Department of Health and Welfare, Iwate Prefecture, Morioka, Iwate Japan; 4 Department of Hygiene and Preventive Medicine, Iwate Medical University, Shiwa-gun, Iwate, Japan; 5 St. Luke’s International University, Tokyo, Japan; 6 Iwate Medical University, Shiwa-gun, Iwate, Japan

**Keywords:** Diseases, Endocrinology, Medical research, Risk factors

## Abstract

The association between incidence of diabetes mellitus (DM) and living conditions has not been studied after natural disasters. We compared the incidence of DM between individuals living in temporary housing (TH) and those living in other types of accommodation (non-TH) five years after the 2011 Great East Japan Earthquake. Longitudinal follow-up was conducted from 2011 to 2015 in a cohort of 7,491 residents of coastal communities in Iwate Prefecture directly impacted by the 2011 disaster (mean age, 61.6 years; men, 36.0%). We calculated the odds ratio of new onset of DM in the TH group (n = 2,372) compared with the non-TH group (n = 5,119) using discrete-time logit models stratified by sex and age classes (64 years or younger and older than 65 years). The TH group showed a significantly higher odds ratio (OR) for DM in men aged 64 years or younger (OR [95% confidence interval (CI)], 1.71 [1.03–2.85]; P-value = 0.040). In women, living conditions were not significantly associated DM. Survivors relocated to TH appeared to be at an increased risk of new onset DM.

## Introduction

In March 11, 2011, a huge earthquake and tsunami struck the coastal areas of northern Honshu, Japan^1,2^. Approximately 22,000 died or were reported missing. Since some survivors’ homes were destroyed, they were placed in evacuation shelters after the disaster, and subsequently moved to prefabricated temporary housing (TH) units several months later. Living conditions in trailer-type TH has been reported to be inconvenient and promote unhealthy life style changes such as less exercise^3,4^ and unhealthy dietary habits^5,6^. Individuals were constrained to live in these conditions for several years after the disaster.

Our previous report has showed that individuals who lived in TH showed an increased body weight 1 to 2 years after the disaster compared with those not living in TH (non-TH) (+ 0.5 kg in both sexes)^7^. Given the need for living in TH for up to 5 years after the disaster, risk factors of cardiovascular diseases might develop. Some studies have determined that the indexes of cardiovascular diseases worsened for survivors after the natural disaster. Studies have reported body weight gain^7^, lipid profile worsening^8^, increased prevalence of metabolic syndrome, and glycosylate hemoglobin elevation^9–11^ for survivors a few years after natural disasters.

Diabetes mellitus (DM) is an important public health concern. The number of patients with DM is rising worldwide and the number of DM patients is currently estimated to be 463 million in 2019^12^. The prevalence of diabetes is two times higher among elderly compared to middle age or young adults^13^. Biological susceptibility is different from sex for the incidence of DM^14^. Due to severe commodities and high mortality resulting from DM, early identification of high-risk individuals is a crucial strategy to prevent development of DM. Some studies have reported that the prevalence of DM and new onset DM increases after natural disasters^15–18^. However, the association between living conditions and the incidence of DM has not been adequately addressed inside affected areas in the aftermath of a natural disaster. In particular, there have been no reports evaluating the incidence of DM in this long-term setting. To prevent from disability driven from DM, we should find high-risk patients earlier after natural disasters.

Therefore, the purpose of our study was to investigate the incidence of DM among individuals living in TH and among individuals living in other types of accommodation (non-TH) in tsunami-affected areas for up to five years after the Great East Japan Earthquake.

## Results

Baseline characteristics between the TH group and the non-TH group are summarized in Table 1. The number of participants was 2,372 in the TH group and 5,119 in the non-TH group. Men in the TH group were significantly younger than those in the non-TH group. Mean ages for women were not different between the two groups. The number of deaths of family members during the disaster was significantly higher in the TH group than in the non-TH group for both sexes. Participants in the TH group had significantly worse conditions at baseline including low physical activity, small number of meals, poor dietary intake, severe economic status, psychological distress, insomnia, than those in the non-TH group in both sexes (*P* < 0.001). Among men in the TH group, a higher prevalence of single and current smokers was observed. Among women in the TH group, there were more single, current smokers, and alcohol consumers.

**Table 1:** Baseline characteristics of participants in the 2011 survey (n = 7,491).

			Men (n = 2,697)				Women (n = 4,794)		
		Missing	TH group (n = 853)	Non-TH group (n = 1844)	*P* value	Missing	TH group (n = 1519)	Non-TH group (n = 3,275)	*P*-value
		n (%)	Mean (SD)/n (%)	Mean (SD)/n (%)		n (%)	Mean (SD)/n (%)	Mean (SD)/n (%)	
**Age**	Age (year)	0 (0.0)	61.9 (13.5)	63.6 (13.6)	0.002	0 (0.0)	60.3 (14.4)	60.9 (13.5)	0.162
**Age classes**	18–44	0 (0.0)	119 (14.0)	221 (12.0)	< 0.001	0 (0.0)	269 (17.7)	461 (14.1)	0.001
	45–54	0 (0.0)	117 (13.7)	163 (8.8)		0 (0.0)	191 (12.6)	395 (12.1)	
	55–64	0 (0.0)	189 (22.2)	436 (23.6)		0 (0.0)	375 (24.7)	975 (29.8)	
	65–75	0 (0.0)	290 (34.0)	651 (35.3)		0 (0.0)	462 (30.4)	992 (30.3)	
	≧ 75	0 (0.0)	138 (16.2)	373 (20.2)		0 (0.0)	222 (14.6)	452 (13.8)	
**Disaster-related experiences**	Death of family members	685 (25.4)	100 (15.7)	94 (6.8)	< 0.001	1,079 (40.0)	179 (15.2)	154 (6.1)	< 0.001
	No death of family members								
**Marital status**	Single (2015)	691 (25.6)	187 (29.4)	253 (18.5)	< 0.001	1,079 (40.0)	440 (37.3)	753 (29.7)	< 0.001
	Married								
**Life style**	Current smokers	0 (0.0)	284 (33.3)	498 (27.0)	0.001	0 (0.0)	117 (7.7)	192 (5.9)	0.016
	Non-current smokers								
	Drinkers	0 (0.0)	562 (65.9)	1,175 (63.7)	0.275	0 (0.0)	239 (15.7)	441 (13.5)	0.036
	Non-drinkers								
	Low physical activity	14 (0.5)	552 (64.9)	1,070 (58.4)	0.001	38 (1.4)	1,083 (71.6)	2,150 (66.3)	< 0.001
	Normal physical activity								
	Small number of meals (< 3 times)	19 (0.7)	80 (9.5)	101 (5.5)	< 0.001	25 (0.9)	92 (6.1)	148 (4.5)	0.022
	Normal number of meals (≥ 3 times)								
	Poor dietary intake	0 (0.0)	402 (47.1)	729 (39.5)	< 0.001	0 (0.0)	518 (34.1)	995 (30.4)	0.010
	Good dietary intake								
**Socioeconomic status**	Severe economic status	9 (0.3)	543 (64.2)	878 (47.7)	< 0.001	11 (0.4)	902 (59.5)	1518 (46.5)	< 0.001
	General economic status								
	Unemployment (2015)	710 (26.3)	322 (51.4)	701 (51.5)	0.977	1,105 (41.0)	760 (64.8)	1665 (66.2)	0.437
	Employment								
**Psychological factors**	Psychological distress	27 (1.0)	346 (41.0)	623 (34.1)	0.001	101 (3.7)	779 (52.6)	1,420 (44.2)	< 0.001
	No psychological distress								
	Insomnia	29 (1.1)	270 (32.0)	412 (22.6)	< 0.001	78 (2.9)	672 (44.9)	1,142 (35.5)	< 0.001
	No insomnia								
**Social factors**	Low level of social network	56 (2.1)	344 (41.1)	767 (42.5)	0.47	102 (3.8)	602 (40.8)	1,288 (40.0)	0.597
	High level of social network								
	Low level of social capital	6 (0.2)	92 (10.8)	193 (10.5)	0.801	16 (0.6)	135 (8.9)	259 (7.9)	0.255
	High level of social capital								
**Cardiovascular risk factors**	Obesity	0 (0.0)	303 (35.5)	678 (36.8)	0.532	10 (0.4)	429 (28.3)	918 (28.1)	0.882
	No obesity								
	Hypertension	0 (0.0)	414 (48.5)	941 (51.0)	0.228	0 (0.0)	638 (42.0)	1,339 (40.9)	0.465
	No hypertension								
	Dyslipidemia	0 (0.0)	307 (36.0)	598 (32.4)	0.069	0 (0.0)	710 (46.7)	1517 (46.3)	0.786
	No dyslipidemia								

Continuous variables indicate mean (standard deviation), categorical variables indicate the number of case (%).

P-values were calculated using the Student t tests for continuous variables and the Chi square test for categorical variables.

*Non-TH* non-temporary housing group, *TH* temporary housing group, *SD* standard deviation.

Table 2 shows the OR of the incidence of DM in discrete-time logit models. In the base models for men (Model 1, age- and living condition-adjusted model), individuals in the TH group had significantly higher OR than those in the non-TH group (OR [95% confidence interval (CI)], 1.34 [1.03–1.84]; P = 0.033). However, this significant difference disappeared in Model 2. Conversely, women in the TH group did not have significantly higher odds ratios in the adjusted models.

**Table 2:** Odds ratios of the incidence of diabetes mellitus in discrete-time logit models.

	Men		Women	
	Model 1	Model 2	Model 1	Model 2
Panel samples	8,171	6,866	15,186	13,151
Number	2,461	1979	4,553	3,680
Event case	221	180	237	193

	OR (95% CI)	OR (95% CI)	OR (95% CI)	OR (95% CI)
2013 (ref = 2012)	0.64 (0.45–0.92)	0.62 (0.41–0.93)	0.58 (0.41–0.81)	0.59 (0.40–0.87)
2014	0.75 (0.52–1.06)	0.74 (0.50–1.10)	0.54 (0.38–0.77)	0.54 (0.36–0.80)
2015	0.55 (0.37–0.81)	0.51 (0.32–0.80)	0.56 (0.39–0.80)	0.75 (0.48–1.16)
TH	1.38 (1.03–1.84)	1.20 (0.85–1.68)	0.95 (0.71–1.28)	0.91 (0.64–1.28)
Age	1.02 (1.01–1.03)	1.02 (1.00–1.04)	1.03 (1.02–1.05)	1.03 (1.01–1.04)
Death of family members		0.86 (0.49–1.52)		1.97 (1.31–2.98)
Single (2015)		0.81 (0.53–1.23)		0.81 (0.59–1.12)
Severe economic status		1.20 (0.86–1.69)		0.70 (0.49–0.98)
Unemployment (2015)		0.94 (0.67–1.33)		1.23 (0.84–1.78)

*OR* odds ratio, *CI* confidence interval, *TH* temporary housing group.

Table 3 shows the ORs of the incidence of DM stratified by sex and age classes. The TH group retained the significantly higher OR for men aged 64 years or younger in Model 2 (OR [95% CI], 1.71 [1.03–2.85]; P = 0.040), but not in men aged 65 years or older (Table 3). In women, the living condition was not significantly associated with DM in either age class (Table 4). Although no significant interactions were found between sex and age classes (P for interaction, 0.293 (≥ 0.20)), substantial differences in characteristics were seen in relation to the development of the incidence of DM by age classes (Supplementary Table S1(A)(B) online).

**Table 3:** Odds ratios of the incidence of diabetes mellitus for men in discrete-time logit models stratified by age class.

Men				
Age class	64 years or younger		65 years or older	
	Model 1	Model 2	Model 1	Model 2
Panel samples	3,643	3,035	4,528	3,831
Number	1,237	888	1,445	1,091
Event case	83	70	138	110

	OR (95% CI)	OR (95% CI)	OR (95% CI)	OR (95% CI)
2013 (ref = 2012)	0.69 (0.38–1.27)	0.65 (0.32–1.28)	0.61 (0.39–0.95)	0.59 (0.35–1.00)
2014	0.89 (0.50–1.58)	0.86 (0.45–1.62)	0.66 (0.42–1.03)	0.67 (0.40–1.11)
2015	0.64 (0.34–1.21)	0.61 (0.30–1.23)	0.48 (0.29–0.81)	0.43 (0.23–0.79)
TH	1.94 (1.24–3.04)	1.71 (1.03–2.85)	1.07 (0.72–1.59)	0.91 (0.56–1.45)
Age	1.05 (1.03–1.08)	1.06 (1.02–1.09)	1.00 (0.97–1.04)	1.00 (0.96–1.04)
Death of family members		0.92 (0.42–2.00)		0.75 (0.32–1.75)
Single (2015)		1.09 (0.62–1.93)		0.67 (0.34–1.30)
Severe economic status		1.08 (0.63–1.86)		1.34 (0.87–2.07)
Unemployment (2015)		0.71 (0.39–1.29)		1.16 (0.74–1.84)

*OR* odds ratio, *CI* confidence interval, *TH* temporary housing group.

**Table 4:** Odds ratios of the incidence of diabetes mellitus for women in discrete-time logit models stratified by age class.

Women				
Age class	64 years or younger		65 years or older	
	Model 1	Model 2	Model 1	Model 2
Panel samples	8,377	7,252	6,809	5,899
Number	2,665	2041	2,125	1639
Event case	102	88	135	105

	OR (95% CI)	OR (95% CI)	OR (95% CI)	OR (95% CI)
2013 (ref = 2012)	0.52 (0.30–0.90)	0.55 (0.31–0.98)	0.61 (0.39–0.96)	0.62 (0.37–1.06)
2014	0.55 (0.32–0.94)	0.53 (0.29–0.94)	0.51 (0.31–0.83)	0.53 (0.30–0.94)
2015	0.54 (0.31–0.92)	0.61 (0.32–1.17)	0.55 (0.34–0.89)	0.85 (0.46–1.58)
TH	1.29 (0.84–1.99)	1.25 (0.77–2.03)	0.75 (0.50–1.14)	0.69 (0.42–1.14)
Age	1.05 (1.02–1.07)	1.04 (1.01–1.07)	1.04 (1.01–1.08)	1.03 (0.99–1.07)
Death of family members		1.14 (0.56–2.33)		2.83 (1.69–4.75)
Single (2015)		0.90 (0.53–1.54)		0.82 (0.54–1.25)
Severe economic status		0.76 (0.46–1.25)		0.65 (0.40–1.05)
Unemployment (2015)		1.41 (0.89–2.24)		1.02 (0.56–1.85)

*OR* odds ratio, *CI* confidence interval, *TH* temporary housing group.

To eliminate the potential prior diagnosis of DM in the baseline survey, we calculated the ORs excluding the participants in the 2012 and 2013 survey (Supplementary Table S2(A)(B) online). In the base models for men aged 64 years or younger, while the TH group retained the significantly higher OR, that significant association disappeared in Model 2 (OR [95% CI], 1.97 [0.95—4.09]; P = 0.070). The results in men aged 65 years or older, and women did not change. In the analysis of all complete cases, we found that the ORs for the development of future DM remained statistically significant for men aged 64 years or younger (Supplementary Table S3(A)(B) online).

## Discussion

We evaluated the incidence of DM in individuals in a tsunami-affected area 5 years after the Great East Japan Earthquake. The ORs of the incidence of DM was significantly higher in men 64 years or younger living in TH than those living in non-TH. This is the first study to determine higher incidence of DM in individuals living in TH for men aged 64 years or younger in the aftermath of the Great East Japan Earthquake.

Several studies have investigated the prevalence and incidence of DM in post-disaster periods. Enber et al. (2016) reported an increased prevalence of DM following the Great East Japan Earthquake in Fukushima^16^. Nomura et al. (2016) and Satoh et al. (2015) showed an increased incidence of DM in individuals who were evacuees than in non-evacuees in Fukushima^15,17^. The aforementioned three studies were conducted in Fukushima where people were affected by the Fukushima Daiichi nuclear power plant explosion. The nature of the disaster was different than in our study area, that is, individuals in the current study were mainly affected by an earthquake and tsunami without any impact from the nuclear accident (which occurred 200 km away, in Fukushima). Most people remained in tsunami-affected towns without evacuating to another inland cities. We determined that living in TH increased the incidence of DM for men aged 64 years or younger. Most studies have examined health problems and higher incidence of diseases after disasters, but these studies featured relatively short follow-up (from 4 to 12 months after the disasters)^9–11^. Although a few studies followed survivors for a longer period (from 3 to 4 years after the disaster) ^16–18^, no study has looked at the longer term incidence of DM. A major strength of our study was that we observed the affected individuals for a long period (up to 5 years) after the disaster.

The mechanisms underlying the relationship between living conditions and higher incidence of DM have not been elucidated in our study. Several predisposing factors are known for developing DM, including genetic diathesis or environmental influences. It is obvious that an increase in body weight resulting from a sedentary life style and unhealthy eating habits are the strongest influences for developing type 2 DM. In the post-disaster setting, environmental changes might have influenced the incidence of DM. Soon after the disaster, survivors experienced food shortages or intake of foods with insufficient nutritional content such as processed food^9^. Thus, at the time of the 2011 survey, high-risk patients for DM might have been masked by improving diet (i.e., they had a lower calorie intake than during the pre-disaster period). In addition, most evacuees moved to TH from 5 months after the disaster. Hikichi et al. reported that survivors who were involuntarily relocated to TH settlements moved closer to fast food outlets/restaurants^19^. Regarding exercise, a previous study has shown that survivors moving to TH reduced their levels of physical activity in Kashima town in Fukushima although physical activity might be lower than in our study due to limitation to go outside with fear for radiation exposure^20^. Environment in these two studies are not completely same as that in our study. But, by improving access to unhealthy sources of food, individuals in TH unintentionally increased body weight by consuming excessive amounts of food and exerting low levels of physical activity. Among East Asians, even a modest increase of body weight was reported to impair adipose tissue insulin sensitivity with less insulin secretory function^21^. We speculated that the same processes increased aggregated blood sugar levels of individuals residing in TH and resulted in a higher-risk of DM during the recovery phase of the disaster (i.e., several months and years later). In the sensitivity analysis excluding participants in the 2012 and 2013 survey, ORs for DM remained significantly higher in participants from the TH group than those from the non-TH group for men in 64 years or younger. Therefore, the influence of high-risk patients was minimum and our results were robust.

Although we could not identify the reasons why men in 64 years or younger had a higher incidence of DM in the TH group, there are some possible explanations. First, the magnitude of body weight change might differ among age classes in a post-disaster period. Initially, our study found that the magnitude of body weight gain was higher for men aged 64 years or younger than for those aged 65 years or older during the survey years (mean changes in body weight [95% CI] in the TH group; aged 64 years or younger, + 1.26 kg [1.17–1.34]; aged 65 years or older, + 0.82 kg [0.73–0.91]). The higher increase in body weight after the disaster might have had a greater impact on the incidence of DM in men aged 64 years or younger. Second, changes in food consumption brought on by an environmental disaster might have an influence to the higher incidence of DM. In the baseline data, although physical activity was not a significant difference between two groups for men 64 years or younger, a fewer number of meals and poor dietary intake was significantly more frequent in the TH group than in the non-TH group (Supplementary Table S1(A)(B) online). Although we were unable to determine the precise amount of food consumption, it is believed that a change in nutrition was a crucial factor in increasing the incidence of DM in men 64 years or younger. For example, conditions in temporary shelters tend to cramped, and discouraged preparation of meals at home^9^. Consequently, people increased their frequency of dining out, which may have led to a higher caloric intake^22^. Third, there might have been a difference in health consciousness by age. According to a nationally representative survey of health consciousness, men aged 64 years or younger tended to be less active with regards to their health maintenance behaviors because they cited being too busy at work^23^. We speculate that men 64 years or younger who had to deal with numerous tasks in the aftermath of the Great East Japan Earthquake might have been too busy to tend to self-care.

Several limitations should be noted for this study. First, some important variables mediating the incidence of DM were not considered, such as the amount of food consumption. Because the target of the survey were individuals living in the disaster-stricken area, starting 6 months after the disaster, we had to limit the survey items to reduce respondent burden. Second, the assessment of DM was conducted without glucose tolerance tests, which might lead to underestimating the incidence of DM. Third, the diagnosis of DM was determined at only one of the four survey waves, which might have led to an underestimation of the risk of DM. We additionally analyzed in the subjects in which we defined the outcome DM at two follow-up assessments (n = 9,249). In stratified analysis by sex and age class, although the TH group showed significantly higher OR for men aged 64 years or younger in Model 1, that higher OR disappeared in Model 2 (OR [95% CI], 1.63 [0.87–3.04]; P = 0.127) (Supplementary Table S4 (A)). The TH group did not have the significantly higher OR in men aged 65 years or older. In women, the living condition was not significantly associated with DM in either age class (Supplementary Table S4 (B)). These results suggest that the measurement error at the present study is small. Fifth, approximately 13.0% to 16% of participants were excluded in the Model which were adjusted for disaster experiences, and marital, economic, and job status (Model 2) because some factors including death of family members, marital status, and employment status were evaluated in the 2015 survey. Participants who were excluded from multivariable analyses due to loss of follow-up might be more likely to develop DM. Sixth, our results may not be generalizable to other disaster-affected areas. The average age in the current study was 61.6 years, which meant the participants were generally older individuals. Our participants may have been particularly healthier or more health-conscious compared to non-participants, which may have led to an underestimation of the incidence of DM.

In the present study, the incidence of DM was significantly higher in individuals re-located to TH, particularly in men aged 64 years or younger, than in disaster victims not living in TH. Survivors relocated to TH appeared to be at an increased risk of DM. These findings imply that an environment characterized by inconvenient living conditions might trigger worse blood sugar levels with unhealthy dietary habits. Because DM leads to severe comorbidities and high mortality, preventive measures are necessary for high-risk citizens and interventions should be devised to address the risk of DM after natural disasters.

## Materials and methods

### Study population

The Research Project for Prospective Investigation of Health Problems Among Survivors of the Great East Japan Earthquake and Tsunami Disaster (RIAS) began after the 2011 Great East Japan Earthquake in northeast coastal area in Iwate prefecture in Japan^24^. The initial survey was conducted in September 2011 (6 months after the disaster) and surveys have been conducted annually thereafter. The details of methodology of the RIAS have been described in a previous report^24^.

**Figure 1:**
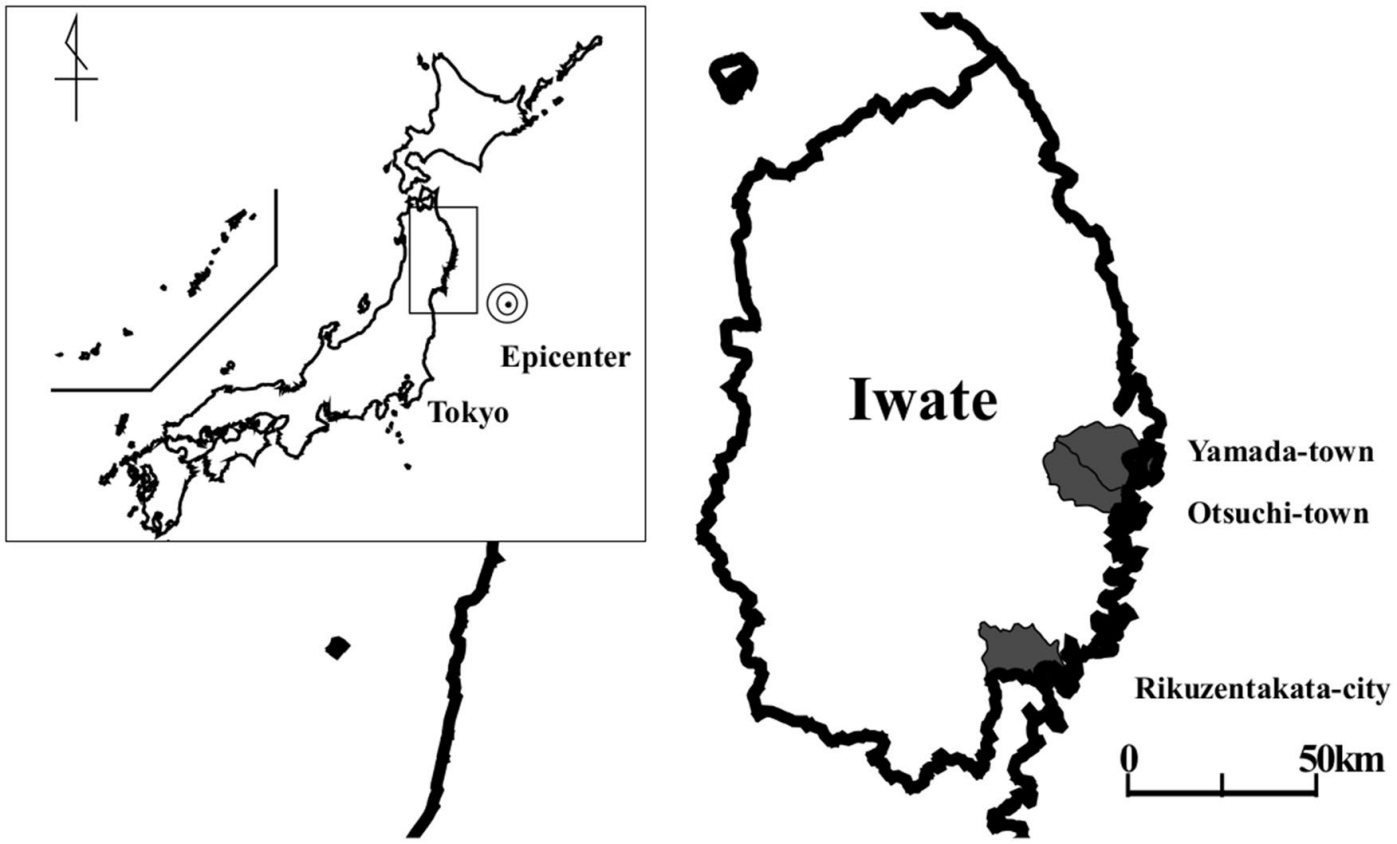
Map of the area surveyed in the present study. The figure shows a map of Japan. The epicenter of the earthquake is marked as a bulls-eye. The municipalities included in our study were Yamada town, Otsuchi town and Rikuzentakata city.

**Figure 2:**
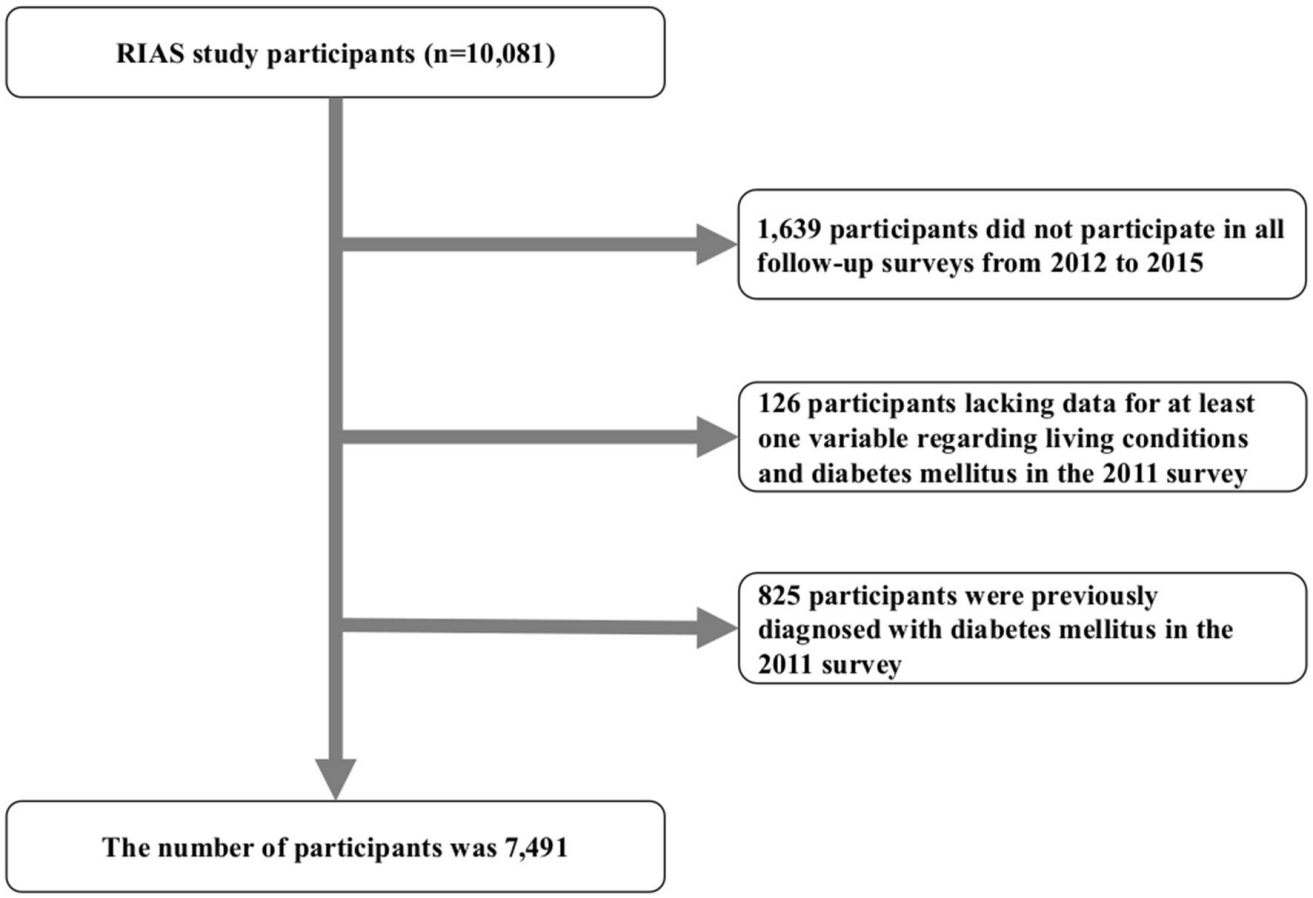
Flow chart used to select participants for the study. The original cohort consisted of 10,081 participants in the baseline survey. After exclusion criteria was met, a total of 7,491 participants remained for the analysis.

A total of 10,081 residents participated in the 2011 survey in Yamada-town, Otsuchi-town, and Rikuzentakata-city, Iwate Prefecture (Fig. 1). After excluding subjects who did not participate in all follow-up surveys from 2012 to 2015 (n = 1,639), those who lacked data for at least one variable regarding living conditions and DM in the 2011 survey (n = 126), and those previously diagnosed with DM in 2011 (n = 825), overall, 7,491 participants, with no evidence of DM at the time of the 2011 survey (mean age, 61.6 years old, men, 36.0%) were analyzed in the study (Fig. 2).

### Living conditions

In the 2011 and 2015 surveys, participants were asked about their current living conditions. Based on their responses, we dichotomized participants into two groups: a TH group (in the 2011 and 2012 surveys; prefabricated TH and shelters: in the 2013, 2014 and 2015 surveys; prefabricated TH) and non-TH (in the 2011 and 2012 surveys; defined as the same house as that during the disaster, a family’s, friend’s, or relative’s house, a new house built after the disaster or rental apartment, or other conditions; in the 2013, 2014 and 2015 surveys: same house as that during the disaster, post-disaster public-funded rental accommodation, relocated to a rental apartment except emergency provisional housings by making use of privately-rented housings, rebuilding a house on the same place as the disaster, a new house built in different place from that before the disaster, a family’s, friend’s, or relative’s house, and other living conditions).

### Outcome measurement

Fasting or non-fasting blood samples were collected from the antecubital vein. Serum samples were centrifuged within an hour of sampling. They were stored at 4 °C were then transferred to the laboratory in Iwate Health Service Association, Morioka, Iwate. Samples were measured on the date of collection or the next date. Glycosylated hemoglobin levels (HbA1c, %) were measured by high-performance liquid chromatography using an automated analyzer (Tosoh HLC-723G7, Japan). HbA1c (Japan Diabetes Society; JDS) was calculated as an equivalent value of the National Glycohemoglobin Standardization Program (NGSP) using the formula HbA1c (NGSP) = HbA1c (JDS values) + 0.4 ^25^. The outcome of this study was the incidence of DM defined as plasma glucose level ≥ 200 mg/dL, plasma HbA1c level (NSGP) ≥ 6.5%, and DM reported in the questionnaire excluding subjects who had already been treated previously, or a combination of these.

### Baseline data and a self-reported questionnaire

We measured body weight using a digital scales stadiometer. The body mass index (BMI; kg/m^2^) was calculated as body weight (kg) divided by height (m^2^). We measured blood pressure (systolic [SBP; mmHg] and diastolic blood pressure [DBP; mmHg]), serum total cholesterol levels (TC; mg/dL) and high-density lipoprotein cholesterol (HDLC; mg/dL).

Participants answered self-report questionnaires to assess demographic characteristics, disaster-related experiences, marital status, life style habits, socioeconomic status, and psychological, social, and cardiovascular risk factors. Most items were assessed in the 2011 survey, but some factors were assessed in the 2015 survey (i.e., the death of family members during the disaster, marital status, and current job status). With regard to disaster-related experiences, the participants were asked: “Did you lose any relatives or family members in the disaster?”. The participants were categorized into two groups (yes or no) according to the death of family members during the disaster. Marital status was assessed in the 2015 survey and classified into two categories: married (married) or single (unmarried, divorced, or widowed). To determine life style, five items were assessed, smoking status, alcohol drinking status, physical activity, the average number of meals per day, and dietary habits. Smoking status was classified as current smokers or non-current smokers. Alcohol drinking status was classified into drinkers vs. non-drinkers. Physical activity was dichotomized into low physical activity (< 23 METs per week) versus normal physical activity (≥ 23 Mets per week). The average number of meals per day several days prior to the survey was classified into two categories: a small number of meals (< 3 times a day) and a normal number of meals (≥ 3 times a day). Dietary intake calculated as good dietary intake and poor dietary intake^26^. To determine socioeconomic status, economic status was assessed and categorized into two groups: severe economic status or general economic status. Current job status was assessed by asking participants the question in the 2015 survey “Are you currently employed and receive income?” Based on their answers, participants were categorized into two groups: unemployment (seeking work or not working) or employment (working). With regard to psychological factors, psychological distress was classified into two categories: psychological distress (scores of 5 − 24) and no psychological distress (score of 0 − 4) using the K6 scale in its Japanese version^27,28^. Insomnia was dichotomized as those with insomnia (scores of 6 − 24) and those with no insomnia (scores of 0 − 5) on the Athens Insomnia Scale (AIS)^29–32^. Regarding social factors, social network was evaluated by the Lubben’s Social Network Scale^33,34^. Social capital was assessed by four questions of the social cohesion regarding the residents’ perceptions of trust in the community and levels of mutual help^26^. We applied a cut-off point of ≤ 9 and classified respondents into two categories: low level and high level of social capital^35^.

Cardiovascular risk factors were assessed by objective indicators and by a self-reported questionnaire including medical history and medication use. Overweight status was calculated as BMI ≥ 25 kg/m^2^. Hypertension was determined by SBP ≥ 140 mmHg, DBP ≥ 90 mmHg, and hypertension reported in a questionnaire or a combination of those. Dyslipidemia was assessed as TC ≥ 220 mg/dL, HDLC < 40 mg/dL, and dyslipidemia reported in the questionnaire or a combination of these^7^. We considered patients with hypertension and dyslipidemia who answered that they had already been successfully treated as without risk factors.

All variables except sex, death of family members, marital status, and current job status were used as time-varying variables.

### Statistical analyses

All statistical analyses performed were stratified by sex considering the differences in biological susceptibility for the incidence of DM. Differences in means/proportions between the TH and the non-TH groups were tested by Student t tests (continuous variables) or the Chi-squared test (categorical variables).

We calculated the odds ratios of new-onset DM using discrete-time logit models in accordance with previous studies^36,37^. We treated each discrete time unit from 2011 to 2015 for each individual as a separate observation. In order to show the incidence of DM after the 2012 survey, the odds ratio (OR) for the incidence of DM from 2013 to 2015 was calculated using 2012 as the reference year. Multivariable models were built introducing a range of confounding variables sequentially: Model 1, adjustment for living conditions and age; Model 2, adding disaster experiences, and marital, economic, and job status. Furthermore, same statistical analyses were performed stratified by sex and age classes: 64 years or younger and 65 years or older. To avoid the influence of potential preexisting DM at the baseline survey, we performed a similar analysis after excluding DM cases in the 2012 and 2013 survey. We also performed the same analysis in complete cases to check the robustness of the results (n = 4,031).

We used Statistical Package for Social Sciences (SPSS) software program, version 25.0 (IBM, Chicago, IL, USA) for all analyses. All p-values < 0.05 were considered statistically significant.

### Ethical considerations

The research plan was approved by the Ethics Committee of Iwate Medical University (approval no. H23-69). All methods were carried out in accordance with STROBE Statement to report our observational study. Informed consent was obtained from all subjects.

## Supplementary information

Supplementary information.

## Data Availability

Data are available from the authors upon reasonable request and with permission of Research Project for Prospective Investigation of Health Problems Among Survivors of the Great East Japan Earthquake and Tsunami Disaster (RIAS) (Iwate Medical University).
