# Supplementary material for: Effect of temporary housing on incidence of diabetes mellitus in survivors of a tsunami-stricken area in 2011 Japan disaster: a serial cross-sectional RIAS study
Source: Sci Rep. 2020 Sep 21;10:15400. doi: 10.1038/s41598-020-71759-4 (PMC7505964; doi:10.1038/s41598-020-71759-4)
Supplement: Supplementary file 1 — Supplementary information. [file 41598_2020_71759_MOESM1_ESM.pdf]

Supplementary Materials

Effect of temporary housing on incidence of diabetes mellitus in survivors of a tsunami-stricken area in 2011 Japan disaster:
A serial cross-sectional RIAS study

Shuko Takahashi; Kozo Tanno; Yuki Yonekura; Haruki Shimoda; Ryohei Sasaki; Kiyomi Sakata; Akira Ogawa; Seiichiro Kobayashi
Correspondence to: shutakahashi-iwt@umin.ac.jp

Supplementary Table S1.
(A) Baseline characteristics of men in the 2011 survey stratified by age classes (n=2,697).
(B) Baseline characteristics of women in the 2011 survey stratified by age classes (n=4,794).

Supplementary Table S2.
(A) Odds ratios of the incidence of diabetes mellitus for men in discrete-time logit models excluding DM events in the 2012 and 2013 survey.
(B) Odds ratios of the incidence of diabetes mellitus for women in discrete-time logit models excluding DM events in the 2012 and 2013 survey.

Supplementary Table S3.
(A) Odds ratios of the incidence of diabetes mellitus for men in discrete-time logit models for all cases.
(B) Odds ratios of the incidence of diabetes mellitus for women in discrete-time logit models for all cases.

Supplementary Table S4.
(A) Odds ratios of the incidence of diabetes mellitus diagnosed two times during surveys for men in discrete-time logit models stratified age classes.
(B) Odds ratios of the incidence of diabetes mellitus diagnosed two times during surveys for women in discrete-time logit models stratified age classes.

Supplementary Table S5.
(A) Baseline characteristics of participants in the 2012 survey (n=7,491).
(B) Baseline characteristics of participants in the 2013 survey (n=7,491).

(C) Baseline characteristics of participants in the 2014 survey (n=7,491).

(D) Baseline characteristics of participants in the 2015 survey (n=7,491).

Supplementary Table S6.

The number of participants and the number of events in categorical variables.

Supplementary Table S1 (A). Baseline characteristics of men in the 2011 survey stratified by age classes (n=2,697)

Men		64 years or younger (n=1245)				65 years or older (n=1452)			
		Missing	TH group (n=425)	Non-TH group (n=820)		Missing	TH group (n=428)	Non-TH group (n=1024)	
		n (%)	Mean (SD)	Mean (SD)	*P* Value	n (%)	Mean (SD)	Mean (SD)	*P* Value
Age	Age (yr)	0 (0.0)	51.1 (10.2)	51.8 (11.3)	0.288	0 (0.0)	72.7 (5.2)	73.1 (5.4)	0.118
Disaster-related experiences	Death of family members	335 (26.9)	57 (18.7)	47 (7.8)	<0.001	350 (28.1)	43 (13.0)	47 (6.1)	<0.001
Marital status	Single (2015)	338 (27.1)	122 (40.1)	164 (27.2)	<0.001	353 (28.4)	65 (19.6)	89 (11.6)	<0.001
Life style	Current smokers	0 (0.0)	202 (47.5)	315 (38.4)	0.002	0 (0.0)	82 (19.2)	183 (17.9)	0.562
	Drinkers	0 (0.0)	295 (69.4)	558 (68.0)	0.623	0 (0.0)	267 (62.4)	617 (60.3)	0.448
	Low physical activity	5 (0.4)	261 (61.4)	465 (57.1)	0.139	9 (0.7)	291 (68.5)	605 (59.4)	0.001
	Small number of meals (<3 times)	5 (0.4)	63 (14.9)	84 (10.3)	0.017	14 (1.1)	17 (4.0)	17 (1.7)	0.007
	Poor dietary intake	0 (0.0)	228 (53.6)	383 (46.7)	0.020	0 (0.0)	174 (40.7)	346 (33.8)	0.013
Socioeconomic status	Severe economic status	2 (0.2)	298 (70.4)	500 (61.0)	0.001	7 (0.6)	245 (57.9)	378 (37.0)	<0.001
	Unemployment (2015)	355 (28.5)	76 (25.7)	132 (22.2)	0.251	355 (28.5)	246 (74.5)	569 (74.2)	0.900
Psychological factors	Psychological distress	9 (0.7)	184 (43.6)	336 (41.3)	0.433	18 (1.4)	162 (38.4)	287 (28.4)	<0.001
	Insomnia	10 (0.8)	148 (35.2)	230 (28.3)	0.013	19 (1.5)	122 (28.8)	182 (18.0)	<0.001
Social factors	Low level of social network	16 (1.3)	182 (43.5)	379 (46.7)	0.287	40 (3.2)	162 (38.6)	388 (39.1)	0.849
	Low level of social capital	2 (0.2)	53 (12.5)	93 (11.4)	0.567	4 (0.3)	39 (9.2)	100 (9.8)	0.711
Cardiovascular risk factors	Obesity	0 (0.0)	158 (37.2)	309 (37.7)	0.861	0 (0.0)	145 (33.9)	369 (36.0)	0.433
	Hypertension	0 (0.0)	155 (36.5)	304 (37.1)	0.834	0 (0.0)	259 (60.5)	637 (62.2)	0.545
	Dyslipidemia	0 (0.0)	166 (39.1)	303 (37.0)	0.467	0 (0.0)	141 (32.9)	295 (28.8)	0.117

Continuous variables indicate mean (standard deviation), categorical variables indicate the number of case (%).
P Values were calculated using the Student t tests for continuous variables and the Chi square test for categorical variables.
Abbreviations: non-TH, non-temporary housing group; TH, temporary housing group; SD, standard deviation.

Supplementary Table S1 (B). Baseline characteristics of women in the 2011 survey stratified by age classes (n=4,794)

Women		64 years or younger (n=2666)				65 years or older (n=2128)			
		Missing	TH group (n=835)	Non-TH group (n=1831)		Missing	TH group (n=684)	Non-TH group (n=1444)	
		n (%)	Mean (SD)	Mean (SD)	*P* Value	n (%)	Mean (SD)	Mean (SD)	*P* Value
Age	Age (yr)	0 (0.0)	50.2 (11.3)	51.8 (10.7)	<0.001	0 (0.0)	72.6 (5.3)	72.4 (5.2)	0.418
Disaster-related experiences	Death of family members	600 (48.2)	94 (14.8)	89 (6.2)	<0.001	479 (38.5)	85.0 (0.2)	65.0 (0.1)	<0.001
Marital status	Single (2015)	600 (48.2)	168 (26.3)	316 (22.1)	0.037	479 (38.5)	272.0 (0.5)	437.0 (0.4)	<0.001
Life style	Current smokers	0 (0.0)	104 (12.5)	180 (9.8)	0.042	0 (0.0)	13.0 (0.0)	12.0 (0.0)	0.032
	Drinkers	0 (0.0)	195 (23.4)	350 (19.1)	0.012	0 (0.0)	44.0 (0.1)	91.0 (0.1)	0.908
	Low physical activity	17 (1.4)	581 (69.7)	1198 (66.0)	0.055	21 (1.7)	502.0 (0.7)	952.0 (0.7)	0.001
	Small number of meals (<3 times)	6 (0.5)	81 (9.7)	131 (7.2)	0.024	19 (1.5)	11.0 (0.0)	17.0 (0.0)	0.409
	Poor dietary intake	0 (0.0)	335 (40.1)	605 (33.0)	<0.001	0 (0.0)	183.0 (0.3)	390.0 (0.3)	0.902
Socioeconomic status	Severe economic status	2 (0.2)	511 (61.3)	992 (54.2)	0.001	9 (0.7)	391.0 (0.6)	526.0 (0.4)	<0.001
	Unemployment (2015)	621 (49.9)	277 (43.8)	708 (50.1)	0.009	484 (38.9)	483.0 (0.9)	957.0 (0.9)	0.111
Psychological factors	Psychological distress	41 (3.3)	454 (55.3)	845 (46.8)	<0.001	60 (4.8)	325.0 (0.5)	575.0 (0.4)	<0.001
	Insomnia	33 (2.7)	357 (43.2)	658 (36.4)	0.001	45 (3.6)	315.0 (0.5)	484.0 (0.3)	<0.001
Social factors	Low level of social network	46 (3.7)	345 (42.3)	804 (44.6)	0.274	56 (4.5)	257.0 (0.4)	484.0 (0.3)	0.033
	Low level of social capital	4 (0.3)	83 (9.9)	159 (8.7)	0.303	12 (1.0)	52.0 (0.1)	100.0 (0.1)	0.570
Cardiovascular risk factors	Obesity	10 (0.8)	224 (26.9)	451 (24.7)	0.228	0 (0.0)	205.0 (0.3)	467.0 (0.3)	0.272
	Hypertension	0 (0.0)	213 (25.5)	467 (25.5)	0.998	0 (0.0)	425.0 (0.6)	872.0 (0.6)	0.441
	Dyslipidemia	0 (0.0)	374 (44.8)	842 (46.0)	0.565	0 (0.0)	336.0 (0.5)	675.0 (0.5)	0.305

Continuous variables indicate mean (standard deviation), categorical variables indicate the number of case (%).

P Values were calculated using the Student t tests for continuous variables and the Chi square test for categorical variables.

Abbreviations: non-TH, non-temporary housing group; TH, temporary housing group; SD, standard deviation.

Supplementary Table S2 (A). Odds ratios of the incidence of diabetes mellitus for men in discrete-time logit models excluding DM events in the 2012 and 2013 survey

Men				
**Age class**	**64 years or younger**		**65 years or older**	
	**Model 1**	**Model 2**	**Model 1**	**Model 2**
**Panel samples**	3583	2988	4417	3749
**Number**	1192	651	1359	1028
**Event case**	38	35	52	47
	**OR (95% CI)**	**OR (95% CI)**	**OR (95% CI)**	**OR (95% CI)**
**TH**	2.01 (1.02 - 3.96)	1.97 (0.95 - 4.09)	1.36 (0.70 - 2.62)	1.59 (0.79 - 3.21)
**Age**	1.06 (1.02 - 1.11)	1.06 (1.01 - 1.11)	0.97 (0.92 - 1.03)	0.98 (0.92 - 1.05)
**Death of family members**		1.41 (0.52 - 3.83)		0.91 (0.27 - 3.08)
**Single (2015)**		0.61 (0.25 - 1.49)		0.41 (0.12 - 1.38)
**Severe economic status**		1.04 (0.43 - 2.48)		0.59 (0.26 - 1.33)
**Unemployment (2015)**		0.90 (0.40 - 2.02)		1.11 (0.57 - 2.20)

Abbreviations: OR, Odds ratio; CI, confidence interval; TH, temporary housing group.

Supplementary Table S2 (B). Odds ratios of the incidence of diabetes mellitus for women in discrete-time logit models excluding DM events in the 2012 and 2013 survey

**Women**				
**Age class**	**64 years or younger**		**65 years or older**	
	**Model 1**	**Model 2**	**Model 1**	**Model 2**
**Panel samples**	8300	7187	6701	5822
**Number**	1891	1605	2042	1581
**Event case**	42	38	52	47
	**OR (95% CI)**	**OR (95% CI)**	**OR (95% CI)**	**OR (95% CI)**
**TH**	1.25 (0.61 - 2.56)	1.36 (0.64 - 2.92)	1.02 (0.53 - 1.95)	0.91 (0.44 - 1.86)
**Age**	1.04 (1.01 - 1.08)	1.03 (0.99 - 1.07)	1.08 (1.02 - 1.13)	1.08 (1.02 - 1.15)
**Death of family members**		0.88 (0.26 - 2.95)		4.87 (2.45 - 9.69)
**Single (2015)**		0.81 (0.35 - 1.88)		0.50 (0.26 - 0.97)
**Severe economic status**		0.53 (0.21 - 1.39)		1.34 (0.58 - 3.13)
**Unemployment (2015)**		1.52 (0.75 - 3.05)		0.80 (0.35 - 1.86)

Abbreviations: OR, Odds ratio; CI, confidence interval; TH, temporary housing group.

Supplementary Table S3 (A). Odds ratios of the incidence of diabetes mellitus for men in discrete-time logit models for all cases

**Men**				
**Age class**	**64 years or younger**		**65 years or older**	
	**Model 1**	**Model 2**	**Model 1**	**Model 2**
**Panel samples**	2464	2464	3254	3254
**Number**	637	637	854	854
**Event case**	52	52	87	87
	**OR (95% CI)**	**OR (95% CI)**	**OR (95% CI)**	**OR (95% CI)**
**2013 (ref= 2012)**	0.52 (0.23–1.17)	0.52 (0.23–1.17)	0.45 (0.25–0.81)	0.45 (0.25–0.82)
**2014**	0.87 (0.43–1.76)	0.87 (0.43–1.78)	0.55 (0.31–0.97)	0.56 (0.31–0.99)
**2015**	0.64 (0.29–1.38)	0.60 (0.26–1.35)	0.45 (0.24–0.83)	0.37 (0.19–0.74)
**TH**	1.78 (1.00–3.16)	1.82 (1.01–3.28)	1.11 (0.68–1.83)	1.11 (0.66–1.84)
**Age**	1.05 (1.02–1.09)	1.05 (1.02–1.09)	0.99 (0.95–1.04)	0.99 (0.95–1.04)
**Death of family members**		1.00 (0.40–2.45)		0.65 (0.23–1.82)
**Single (2015)**		0.93 (0.47–1.84)		0.44 (0.18–1.12)
**Severe economic status**		1.16 (0.62–2.15)		1.44 (0.89–2.32)
**Unemployment (2015)**		0.75 (0.38–1.49)		1.62 (0.92–2.84)

Abbreviations: OR, Odds ratio; CI, confidence interval; TH, temporary housing group.

Supplementary Table S3 (B). Odds ratios of the incidence of diabetes mellitus for women in discrete-time logit models for all cases

Women				
Age class	64 years or younger		65 years or older	
	Model 1	Model 2	Model 1	Model 2
Panel samples	6061	6061	5005	5005
Number	1545	1545	1290	1290
Event case	64	64	92	92
	OR (95% CI)	OR (95% CI)	OR (95% CI)	OR (95% CI)
2013 (ref= 2012)	0.55 (0.29–1.05)	0.55 (0.29–1.05)	0.52 (0.29–0.93)	0.51 (0.29–0.91)
2014	0.39 (0.19–0.79)	0.39 (0.19–0.80)	0.48 (0.26–0.87)	0.47 (0.26–0.86)
2015	0.40 (0.20–0.81)	0.46 (0.21–1.00)	0.64 (0.37–1.11)	0.83 (0.43–1.60)
TH	1.22 (0.70–2.12)	1.24 (0.71–2.18)	0.76 (0.46–1.25)	0.73 (0.43–1.23)
Age	1.06 (1.03–1.10)	1.05 (1.02–1.10)	1.03 (0.98–1.07)	1.03 (0.98–1.07)
Death of family members		1.17 (0.52–2.63)		2.74 (1.56–4.80)
Single (2015)		1.07 (0.59–1.95)		0.87 (0.55–1.37)
Severe economic status		0.84 (0.48–1.47)		0.65 (0.39–1.10)
Unemployment (2015)		1.49 (0.85–2.59)		0.98 (0.53–1.84)

Abbreviations: OR, Odds ratio; CI, confidence interval; TH, temporary housing group.

Supplementary Table S4 (A). Odds ratios of the incidence of diabetes mellitus diagnosed two times during surveys for men in discrete-time logit models stratified age classes

**Men**				
**Age class**	**64 years or younger**		**65 years or older**	
	**Model 1**	**Model 2**	**Model 1**	**Model 2**
**Panel samples**	3643	2393	3466	3044
**Number**	1134	904	1408	1127
**Event case**	55	48	107	89
	**OR (95% CI)**	**OR (95% CI)**	**OR (95% CI)**	**OR (95% CI)**
**2014 (ref= 2013)**	0.45 (0.22 - 0.92)	0.44 (0.20 - 0.97)	0.48 (0.30 - 0.77)	0.49 (0.29 - 0.84)
**2015**	0.77 (0.42 - 1.43)	0.70 (0.34 - 1.41)	0.54 (0.34 - 0.86)	0.59 (0.32 - 1.09)
**TH**	1.80 (1.03 - 3.14)	1.63 (0.87 - 3.04)	0.64 (0.37 - 1.11)	0.62 (0.33 - 1.16)
**Age**	1.06 (1.02 - 1.09)	1.06 (1.02 - 1.11)	1.02 (0.98 - 1.06)	1.02 (0.98 - 1.07)
**Death of family members**		1.09 (0.44 - 2.67)		0.65 (0.25 - 1.65)
**Single (2015)**		1.19 (0.61 - 2.32)		1.40 (0.78 - 2.51)
**Severe economic status**		1.37 (0.66 - 2.83)		0.95 (0.56 - 1.62)
**Unemployment (2015)**		1.09 (0.56 - 2.10)		0.95 (0.58 - 1.56)

Abbreviations: OR, Odds ratio; CI, confidence interval; TH, temporary housing group.

Supplementary Table S4 (B). Odds ratios of the incidence of diabetes mellitus diagnosed two times during surveys for women in discrete-time logit models stratified age classes

Women				
**Age class**	**64 years or younger**		**65 years or older**	
	**Model 1**	**Model 2**	**Model 1**	**Model 2**
**Panel samples**	6210	5602	5138	4602
**Number**	2485	2061	2036	1607
**Event case**	69	58	94	73
	**OR (95% CI)**	**OR (95% CI)**	**OR (95% CI)**	**OR (95% CI)**
**2014 (ref= 2013)**	0.52 (0.29 - 0.92)	0.55 (0.30 - 1.03)	0.47 (0.28 - 0.78)	0.41 (0.22 - 0.75)
**2015**	0.45 (0.25 - 0.82)	0.42 (0.20 - 0.87)	0.50 (0.30 - 0.83)	0.48 (0.25 - 0.92)
**TH**	1.03 (0.58 - 1.81)	1.00 (0.53 - 1.88)	0.70 (0.41 - 1.17)	0.42 (0.21 - 0.84)
**Age**	1.03 (1.00 - 1.06)	1.03 (1.00 - 1.07)	1.02 (0.98 - 1.06)	1.04 (0.99 - 1.09)
**Death of family members**		1.29 (0.54 - 3.08)		1.43 (0.67 - 3.06)
**Single (2015)**		0.72 (0.36 - 1.44)		1.12 (0.68 - 1.82)
**Severe economic status**		1.04 (0.57 - 1.91)		1.22 (0.69 - 2.16)
**Unemployment (2015)**		1.20 (0.69 - 2.09)		1.27 (0.57 - 2.81)

Abbreviations: OR, Odds ratio; CI, confidence interval; TH, temporary housing group.

Supplementary Table S5 (A). Baseline characteristics of participants in the 2012 survey (n=7,491)

		Men (n=2697)				Women (n=4794)			
		Missing	TH group (n=853)	Non-TH group (n=1844)		Missing	TH group (n=1519)	Non-TH group (n=3275)	
		n (%)	Mean (SD)/ n (%)	Mean (SD)/ n (%)	*P* Value	n (%)	Mean (SD)/ n (%)	Mean (SD)/ n (%)	*P*-value
**Age**	**Age (yr)**	392 (14.5)	62.7 (13.4)	65.1 (13.4)	<0.001	589 (16.5)	61.4 (14.2)	62.0 (13.2)	0.203
**Disaster-related experiences**	**Death of family members**	945 (35.0)	87 (16.1)	83 (6.8)	<0.001	1494 (41.7)	157 (15.6)	144 (6.3)	<0.001
**Marital status**	**Single (2015)**	950 (35.2)	162 (30.1)	215 (17.8)	<0.001	1495 (41.8)	385 (38.3)	655 (28.6)	<0.001
**Life style**	**Current smokers**	392 (14.5)	228 (31.8)	393 (24.8)	<0.001	589 (16.5)	87 (6.7)	150 (5.1)	0.037
	**Drinkers**	392 (14.5)	481 (67.0)	987 (62.2)	0.027	589 (16.5)	185 (14.4)	415 (14.2)	0.918
	**Low physical activity**	398 (14.8)	271 (37.8)	462 (29.2)	<0.001	615 (17.2)	407 (31.8)	673 (23.2)	0.001
	**Small number of meals (<3 times)**	413 (15.3)	67 (9.4)	85 (5.4)	<0.001	627 (17.5)	73 (5.7)	131 (4.5)	0.103
	**Poor dietary intake**	392 (14.5)	392 (54.6)	696 (43.9)	<0.001	587 (16.4)	450 (34.9)	912 (31.3)	0.021
**Socioeconomic status**	**Severe economic status**	399 (14.8)	403 (56.3)	643 (40.6)	<0.001	602 (16.8)	698 (54.4)	1109 (38.1)	<0.001
	**Unemployment (2015)**	961 (35.6)	278 (52.3)	633 (52.6)	0.902	1515 (42.3)	671 (67.1)	1524 (66.9)	0.898
**Psychological factors**	**Psychological distress**	418 (15.5)	228 (32.2)	350 (22.3)	<0.001	652 (18.2)	545 (42.9)	949 (33.0)	<0.001
	**Insomnia**	421 (15.6)	166 (23.4)	253 (16.1)	<0.001	649 (18.1)	434 (34.3)	751 (26.1)	<0.001
**Social factors**	**Low level of social network**	434 (16.1)	322 (45.5)	688 (44.2)	0.556	672 (18.8)	557 (43.9)	1265 (44.3)	0.813
	**Low level of social capital**	401 (14.9)	76 (10.6)	173 (10.9)	0.835	610 (17.0)	126 (9.8)	275 (9.5)	0.721
**Cardiovascular risk factors**	**Obesity**	392 (14.5)	283 (39.4)	557 (35.1)	0.046	596 (16.6)	399 (31.0)	822 (28.2)	0.069
	**Hypertension**	395 (14.6)	365 (50.9)	852 (53.8)	0.205	599 (16.7)	564 (43.9)	1221 (41.9)	0.232
	**Dyslipidemia**	395 (14.6)	256 (35.7)	490 (30.9)	0.023	599 (16.7)	605 (47.1)	1385 (47.6)	0.783

Continuous variables indicate mean (standard deviation), categorical variables indicate the number of case (%).

P-values were calculated using the Student t tests for continuous variables and the Chi square test for categorical variables.

Abbreviations: non-TH, non-temporary housing group; TH, temporary housing group; SD, standard deviation.

Supplementary Table S5 (B). Baseline characteristics of participants in the 2013 survey (n=7,491)

		Men (n=2697)				Women (n=4794)			
		Missing	TH group (n=853)	Non-TH group (n=1844)		Missing	TH group (n=1519)	Non-TH group (n=3275)	
		n (%)	Mean (SD)/ n (%)	Mean (SD)/ n (%)	*P* Value	n (%)	Mean (SD)/ n (%)	Mean (SD)/ n (%)	*P*-value
**Age**	**Age (yr)**	550 (20.4)	64.0 (13.0)	66.7 (12.6)	<0.001	867 (24.2)	63.0 (13.8)	63.4 (12.8)	0.381
**Disaster-related experiences**	**Death of family members**	954 (35.4)	76 (16.0)	87 (6.9)	<0.001	1482 (41.4)	141 (15.6)	156 (6.5)	<0.001
**Marital status**	**Single (2015)**	958 (35.5)	135 (28.5)	232 (18.3)	<0.001	1483 (41.4)	363 (40.1)	680 (28.3)	<0.001
**Life style**	**Current smokers**	550 (20.4)	393 (67.4)	939 (60.0)	<0.001	867 (24.2)	71 (6.7)	127 (4.4)	0.004
	**Drinkers**	550 (20.4)	393 (67.4)	939 (60.0)	0.002	867 (24.2)	0 (92400.0)	0 (247200.0)	0.619
	**Low physical activity**	551 (20.4)	155 (26.6)	285 (18.2)	<0.001	854 (23.9)	207 (19.3)	362 (12.6)	<0.001
	**Small number of meals (<3 times)**	572 (21.2)	49 (8.5)	87 (5.6)	0.014	912 (25.5)	56 (5.3)	105 (3.7)	0.024
	**Poor dietary intake**	556 (20.6)	270 (46.2)	604 (38.8)	0.002	869 (24.3)	331 (31.1)	733 (25.6)	0.001
**Socioeconomic status**	**Severe economic status**	545 (20.2)	340 (58.1)	585 (37.3)	<0.001	849 (23.7)	597 (55.8)	983 (34.2)	<0.001
	**Unemployment (2015)**	972 (36.0)	234 (50.4)	673 (53.4)	0.278	1501 (41.9)	591 (65.7)	1618 (67.6)	0.315
**Psychological factors**	**Psychological distress**	567 (21.0)	160 (27.9)	331 (21.3)	0.001	895 (25.0)	429 (40.9)	831 (29.1)	<0.001
	**Insomnia**	577 (21.4)	134 (23.1)	256 (16.6)	0.001	888 (24.8)	353 (33.4)	726 (25.5)	<0.001
**Social factors**	**Low level of social network**	581 (21.5)	214 (37.3)	534 (34.6)	0.257	907 (25.3)	410 (38.9)	1014 (35.8)	0.074
	**Low level of social capital**	561 (20.8)	62 (10.7)	188 (12.1)	0.355	884 (24.7)	101 (9.5)	318 (11.2)	0.150
**Cardiovascular risk factors**	**Obesity**	550 (20.4)	241 (41.3)	519 (33.2)	<0.001	872 (24.4)	324 (30.5)	811 (28.4)	0.187
	**Hypertension**	545 (20.2)	304 (52.0)	871 (55.6)	0.134	845 (23.6)	489 (45.7)	1207 (41.9)	0.033
	**Dyslipidemia**	545 (20.2)	231 (39.5)	564 (36.0)	0.135	845 (23.6)	576 (53.8)	1563 (54.3)	0.797

Continuous variables indicate mean (standard deviation), categorical variables indicate the number of case (%).

P-values were calculated using the Student t tests for continuous variables and the Chi square test for categorical variables.

Abbreviations: non-TH, non-temporary housing group; TH, temporary housing group; SD, standard deviation.

Supplementary Table S5 (C). Baseline characteristics of participants in the 2014 survey (n=7,491)

		Men (n=2697)				Women (n=4794)			
		**Missing**	**TH group (n=853)**	**Non-TH group (n=1844)**		**Missing**	**TH group (n=1519)**	**Non-TH group (n=3275)**	
		**n (%)**	**Mean (SD)/ n (%)**	**Mean (SD)/ n (%)**	***P* Value**	**n (%)**	**Mean (SD)/ n (%)**	**Mean (SD)/ n (%)**	***P*-value**
**Age**	**Age (yr)**	654 (24.2)	64.0 (13.0)	66.7 (12.6)	<0.001	1060 (29.6)	63.0 (13.8)	63.4 (12.8)	0.381
**Disaster-related experiences**	**Death of family members**	896 (33.2)	66 (15.9)	101 (7.3)	<0.001	1455 (40.6)	120 (15.4)	180 (7.0)	<0.001
**Marital status**	**Single (2015)**	901 (33.4)	127 (30.6)	258 (18.7)	<0.001	1455 (40.6)	327 (41.9)	729 (28.5)	<0.001
**Life style**	**Current smokers**	654 (24.2)	134 (28.6)	341 (21.7)	0.002	1060 (29.6)	52 (6.0)	135 (4.7)	0.127
	**Drinkers**	654 (24.2)	297 (63.5)	966 (61.3)	0.405	1060 (29.6)	113 (13.0)	387 (13.5)	0.725
	**Low physical activity**	629 (23.3)	112 (23.6)	268 (16.8)	0.001	1036 (28.9)	152 (17.4)	306 (10.6)	<0.001
	**Small number of meals (<3 times)**	643 (23.8)	48 (10.3)	86 (5.4)	<0.001	1064 (29.7)	49 (5.7)	109 (3.8)	0.018
	**Poor dietary intake**	658 (24.4)	212 (45.5)	611 (38.8)	0.010	1094 (30.6)	264 (30.5)	772 (27.2)	0.059
**Socioeconomic status**	**Severe economic status**	628 (23.3)	266 (56.1)	617 (38.7)	<0.001	1035 (28.9)	484 (55.3)	1018 (35.3)	<0.001
	**Unemployment (2015)**	919 (34.1)	198 (48.9)	716 (52.1)	0.249	1475 (41.2)	518 (66.7)	1697 (66.8)	0.962
**Psychological factors**	**Psychological distress**	641 (23.8)	133 (28.2)	275 (17.4)	<0.001	1078 (30.1)	292 (33.7)	761 (26.7)	<0.001
	**Insomnia**	652 (24.2)	112 (24.0)	228 (14.4)	<0.001	1076 (30.1)	258 (29.9)	651 (22.8)	<0.001
**Social factors**	**Low level of social network**	675 (25.0)	183 (39.6)	521 (33.4)	0.014	1110 (31.0)	322 (37.8)	954 (33.7)	0.025
	**Low level of social capital**	634 (23.5)	69 (14.6)	188 (11.8)	0.110	1054 (29.4)	99 (11.4)	282 (9.8)	0.184
**Cardiovascular risk factors**	**Obesity**	654 (24.2)	181 (38.7)	538 (34.2)	0.072	1068 (29.8)	283 (32.8)	823 (28.8)	0.024
	**Hypertension**	627 (23.2)	223 (46.9)	879 (55.1)	0.002	1031 (28.8)	410 (46.9)	1258 (43.6)	0.085
	**Dyslipidemia**	627 (23.2)	198 (41.7)	605 (37.9)	0.141	1031 (28.8)	486 (55.5)	1535 (53.2)	0.214

Continuous variables indicate mean (standard deviation), categorical variables indicate the number of case (%).
P-values were calculated using the Student t tests for continuous variables and the Chi square test for categorical variables.

Abbreviations: non-TH, non-temporary housing group; TH, temporary housing group; SD, standard deviation.

Supplementary Table S5 (D). Baseline characteristics of participants in the 2015 survey (n=7,491)

		Men (n=2697)				Women (n=4794)			
		**Missing**	**TH group (n=853)**	**Non-TH group (n=1844)**		**Missing**	**TH group (n=1519)**	**Non-TH group (n=3275)**	
		**n (%)**	**Mean (SD)/ n (%)**	**Mean (SD)/ n (%)**	***P* Value**	**n (%)**	**Mean (SD)/ n (%)**	**Mean (SD)/ n (%)**	***P*-value**
**Age**	**Age (yr)**	713 (26.4)	64.8 (12.4)	68.1 (12.4)	<0.001	1127 (31.5)	65.0 (13.3)	65.1 (12.2)	0.820
**Disaster-related experiences**	**Death of family members**	689 (25.5)	67 (17.9)	126 (7.7)	<0.001	1093 (30.5)	110 (15.2)	220 (7.4)	<0.001
**Marital status**	**Single (2015)**	695 (25.8)	126 (33.7)	313 (19.2)	<0.001	1093 (30.5)	312 (43.2)	877 (29.4)	<0.001
**Life style**	**Current smokers**	713 (26.4)	99 (26.7)	356 (22.1)	0.057	1127 (31.5)	45 (6.3)	126 (4.3)	0.021
	**Drinkers**	713 (26.4)	236 (63.6)	976 (60.5)	0.269	1127 (31.5)	109 (15.3)	390 (13.2)	0.150
	**Low physical activity**	897 (33.3)	82 (23.8)	228 (15.7)	<0.001	1461 (40.8)	120 (18.2)	249 (9.3)	<0.001
	**Small number of meals (<3 times)**	713 (26.4)	45 (12.1)	83 (5.1)	<0.001	1118 (31.2)	36 (5.0)	95 (3.2)	0.018
	**Poor dietary intake**	719 (26.7)	191 (51.5)	596 (37.1)	<0.001	1128 (31.5)	212 (29.8)	714 (24.2)	0.002
**Socioeconomic status**	**Severe economic status**	694 (25.7)	365 (97.3)	1568 (96.3)	0.333	1096 (30.6)	709 (98.1)	2885 (97.0)	0.112
	**Unemployment (2015)**	714 (26.5)	178 (48.8)	841 (52.0)	0.268	1119 (31.3)	472 (65.8)	1941 (65.6)	0.915
**Psychological factors**	**Psychological distress**	703 (26.1)	100 (26.8)	295 (18.2)	<0.001	1128 (31.5)	254 (35.7)	788 (26.7)	<0.001
	**Insomnia**	710 (26.3)	71 (19.1)	231 (14.3)	0.019	1127 (31.5)	217 (30.3)	651 (22.1)	<0.001
**Social factors**	**Low level of social network**	936 (34.7)	142 (42.1)	478 (33.6)	0.003	1533 (42.8)	246 (38.5)	891 (34.0)	0.032
	**Low level of social capital**	695 (25.8)	48 (12.8)	202 (12.4)	0.822	1130 (31.6)	73 (10.2)	291 (9.9)	0.795
**Cardiovascular risk factors**	**Obesity**	713 (26.4)	157 (42.3)	554 (34.3)	0.004	1131 (31.6)	215 (30.1)	848 (28.8)	0.474
	**Hypertension**	687 (25.5)	182 (48.4)	931 (57.0)	0.003	1091 (30.5)	345 (47.7)	1315 (44.1)	0.082
	**Dyslipidemia**	687 (25.5)	161 (42.8)	577 (35.3)	0.006	1091 (30.5)	366 (50.6)	1486 (49.9)	0.715

Continuous variables indicate mean (standard deviation), categorical variables indicate the number of case (%).
P-values were calculated using the Student t tests for continuous variables and the Chi square test for categorical variables.

Abbreviations: non-TH, non-temporary housing group; TH, temporary housing group; SD, standard deviation

Supplementary Table S6. The number of participants and the number of events in categorical variables

		Men (n=2698)			Women (n=4794)		
		Missing	Participants (n=2698)	Event (n=222)	Missing	Participants (n=4794)	Event (n=238)
		n (%)	n	n	n (%)	n	n
**Living conditions**	**Temporary housing group**	0 (0.0)	853	81	0 (0.0)	1519	77
	**Non-temporary housing group**		1844	141		3275	161
**Disaster influence**	**Death of family members**	685 (25.4)	194	14	1079 (22.5)	333	29
	**No death of family members**		1818	167		3382	167
**Marital status**	**Single (2015)**	691 (25.6)	440	30	1079 (22.5)	1193	63
	**Married**		1566	151		2522	133
**Life style**	**Current smokers**	0 (0.0)	782	69	0 (0.0)	309	10
	**Non-current smokers**		1915	153		4485	228
	**Drinkers**	0 (0.0)	1737	145	0 (0.0)	680	29
	**Non-drinkers**		960	77		4114	209
	**Low physical activity**	14 (0.5)	1622	132	38 (0.8)	3233	170
	**Normal physical activity**		1061	88		1523	68
	**Small number of meals (< 3 times)**	19 (0.7)	181	8	25 (0.5)	240	2
	**Normal number of meals (≥3 times)**		2497	213		4529	235
	**Poor dietary intake**	0 (0.0)	1131	80	0 (0.0)	1513	64
	**Good dietary intake**		1566	142		3281	174
**Socioeconomic status**	**Severe economic status**	9 (0.3)	1421	124	11 (0.2)	2420	111
	**General economic status**		1267	98		2363	126
	**Unemployment (2015)**	710 (26.3)	1023	101	1105 (23.0)	2425	148
	**Employment**		964	80		1264	47
**Psychological factors**	**Psychological distress**	27 (1.0)	969	76	101 (2.1)	2199	99
	**No psychological distress**		1701	143		2494	133
	**Insomnia**	29 (1.1)	682	52	78 (1.6)	1814	86
	**No insomnia**		1986	169		2902	145
**Social factors**	**Low level of social network**	56 (2.1)	1111	85	102 (2.1)	1890	99
	**High level of social network**		1530	131		2802	134

	**Low level of social capital**	6 (0.2)	285	25	16 (0.3)	394	22
	**High level of social capital**		2406	195		4384	214
**Cardiovascular risk factors**	**Obesity**	0 (0.0)	981	87	10 (0.2)	1347	98
	**No Obesity**		1716	135		3437	140
	**Hypertension**	0 (0.0)	1355	144	0 (0.0)	1977	154
	**No hypertension**		1342	78		2817	84
	**Dyslipidemia**	0 (0.0)	905	87	0 (0.0)	2227	142
	**No dyslipidemia**		1792	135		2567	96

Variables indicate the number of case.
